# Does resection after neoadjuvant chemotherapy of docetaxel, oxaliplatin, and S-1 (DOS regimen) benefit for gastric cancer patients with single non-curable factor? a multicenter, prospective cohort study (Neo-REGATTA)

**DOI:** 10.1186/s12885-023-10773-x

**Published:** 2023-04-04

**Authors:** Yuehong Cui, Yiyi Yu, Song Zheng, Jie’er Ying, Yi’an Du, Yan Wang, Xuefei Wang, Zhenbin Shen, Fenglin Liu, Minzhi Lv, Yihong Sun, Tianshu Liu

**Affiliations:** 1 Dept of Medical oncology, Zhongshan Hospital, Fudan University, Shanghai, China; 2 Dept of Medical oncology, Hangzhou first people’s Hospital, Hangzhou city, Zhejiang Province China; 3 Dept of Medical oncology, Zhejiang Cancer Hospital, Hangzhou city, Zhejiang Province China; 4 Dept of General Surgery, Zhongshan Hospital, Fudan University, Shanghai, China; 5 Cancer center, Zhongshan Hospital, Fudan University, Shanghai, China; 6 Dept of Biostatistics, Zhongshan Hospital, Fudan University, Shanghai, China

**Keywords:** Stomach neoplasms, Adenocarcinoma, Oligometastasis, Radical resection, Perioperative chemotherapy, Efficacy, Safety

## Abstract

**Background:**

The Neo-REGATTA study evaluated the effectiveness and safety of Docetaxel, oxaliplatin, and S-1 (DOS regimen) followed by radical resection vs. chemotherapy in advanced gastric adenocarcinoma patients with single non-curable factor.

**Methods:**

This cohort study prospectively enrolled advanced gastric adenocarcinoma patients with single non-curable factor between November 2017 and June 2021. Patients without progression after four cycles of DOS were divided into resection group and chemotherapy group. The outcomes included overall survival (OS), progression-free survival (PFS) and safety. Effectiveness analysis was also performed by propensity score matching (PSM).

**Results:**

A total of 73 patients were enrolled and 13 patients were withdrawn due to disease progression after 4 cycles of DOS. Afterwards, 35 and 25 participants were in the resection and chemotherapy groups, respectively. After a median follow-up time of 30.0 months, the median PFS and OS were 9.0 months, and 18.0 months for the chemotherapy group, but not reached in the resection group. After PSM, 19 matched participants were in each group, and the median PFS and OS were longer in resection group than that in chemotherapy group. The most common grade 3 or 4 adverse events both in the resection group and chemotherapy groups were neutropenia (5.7%, 8.0%) and leukopenia (5.7%, 8.0%).

**Conclusions:**

Radical resection might provide survival benefit compared with continuous chemotherapy alone in advanced gastric adenocarcinoma patients who had a disease control after DOS, with a good safety profile.

**Trial registration:**

The study protocol was registered on ClinicalTrial.gov (NCT03001726, 23/12/2016).

**Supplementary Information:**

The online version contains supplementary material available at 10.1186/s12885-023-10773-x.

## Background

Gastric cancer includes tumors of the non-cardia and the sub-cardia (Siewert type III), with the center starting 2–5 cm below the esophagogastric junction [1, 2]. Patients often present with nonspecific symptoms that may include anorexia, weight loss, abdominal pain, dyspepsia, vomiting, and early satiety, thus diagnosis is mostly performed in the advanced stage in many cases [1, 3]. The management of advanced gastric cancer is multidisciplinary and includes surgery, systemic therapies, and radiotherapy [1, 3]. Nevertheless, the median overall survival (OS) is about 11.2 months in patients with locally advanced unresectable and advanced metastatic gastric cancers [4].

It is considered that patients with a single non-curable factor are most likely to obtain a survival benefit from a surgically reduced tumor burden. Indeed, several subgroup analyses of clinical trials [5–7] and retrospective patient cohorts [8–16] have exhibited potential benefits, while others reported discrepant data [17–21]. A pilot phase II trial showed that conventional chemotherapy followed by radical resection prolonged the median survival to 29.8 months in advanced gastric adenocarcinoma patients with limited metastasis of para-aortic lymph nodes [22]. For cases of cytology-positive peritoneal lavage fluids (CY1), with the help of radical resection followed by S-1, the median recurrence-free survival (RFS) and OS reached 12.5 months and 23.5 months in the CCOG0301 trial [23]. However, a phase III randomized controlled trial (REGATTA) reported that gastrectomy followed by palliative chemotherapy (S-1 plus cisplatin) showed no survival benefit compared to chemotherapy alone in advanced gastric cancer with a single non-curable factor [24], which inferred that chemotherapy alone might remain the mainstay of care in advanced gastric cancer with a single non-curable factor [24].

Recently, a phase III clinical trial (PRODIGY) enrolled patients with resectable locally advanced gastric cancer who were randomized to D2 gastrectomy plus S-1 adjuvant therapy, or preoperative neoadjuvant Docetaxel, oxaliplatin, and S-1 (DOS) regimen plus D2 gastrectomy then S-1 adjuvant therapy. They found neoadjuvant DOS, as part of perioperative chemotherapy, improved PFS of patients with advanced gastric cancer, and treatments were well tolerated [25]. Other studies also showed that the preoperative DOS regimen is effective and safe in patients with locally advanced gastric adenocarcinoma [26] and metastatic gastric cancer [27, 28].

Due to the favorable response and tolerable toxicity for the preoperative DOS regimen, we hypothesized that radical resection after DOS treatment provide survival benefit compared with continuous chemotherapy in advanced gastric adenocarcinoma patients with a single non-curable factor. Thus, the present study aimed to investigate the effectiveness and safety of the DOS regimen followed by radical resection or continuous chemotherapy in gastric adenocarcinoma patients with single non-curable factor.

## Methods

### Study design and participants

At beginning, the Neo-REGATTA study was designed to be a multicenter, randomized, controlled phase III trial to evaluate the efficacy and safety of perioperative DOS chemotherapy in gastric adenocarcinoma patients with single non-curable factor (exact definition presented below) in combination with radical resection, including the primary and metastatic lesions. Unfortunately, enrollment was very difficult because the participants would not comply with the results of randomization. Therefore, after discussion with biostatisticians, the protocol was amended to be a multicenter, prospective cohort study. Patients diagnosed with gastric adenocarcinoma with single non-curable factor from November 2017 to June 2021 in three hospitals were eligible.

The inclusive and exclusive criteria in detail were seen in Supplementary File 1. Single non-curable factor was defined as (1) para-aortic lymph node metastasis: located above celiac trunk or below the inferior mesenteric artery (diameter ≥ 1 cm); (2) peritoneum metastasis, with limited lesions in the peritoneum above the transverse colon, including the diaphragm, mesentery, and greater omentum (potentially resectable metastatic lesions evaluated by laparoscopy); (3) liver metastasis with one lesion or limited in one lobe, potentially resectable, and sufficient liver function after surgery; (4) unilateral or bilateral Krukenberg’s tumor (ovarian metastasis) diagnosed by enhanced CT or laparoscopic exploration.

The study protocol was registered on ClinicalTrial.gov (# NCT03001726). The study complied with the Declaration of Helsinki, the principles of Good Clinical Practice guidelines, and the Data Protection Act. The study was approved by the ethics committee of Zhongshan Hospital affiliated with Fudan University. The participants provided written informed consent before enrollment.

### Procedures

After diagnosis, qualified participants received four cycles of DOS (details shown below) and underwent evaluation and discussion by multi-disciplinary team (MDT). For patients evaluated as disease progression were withdrawn from this study and continued second-line therapy under the patient’s willingness. For those patients well-responded to the DOS regimen, patients were enrolled to perform radical surgery or continuous chemotherapy of DOS.

For radical surgery cohort, D2 surgery was recommended combined with R0 resection of metastatic site. After surgery, post-operative chemotherapy of DOS were recommended maximal to 4 cycles. For continuous chemotherapy cohort, four cycles of DOS followed by single S-1 maintenance.

Resection cohort: Surgery was scheduled within 2–4 weeks after the end of preoperative DOS chemotherapy (docetaxel 40 mg/m^2^, iv over 1 h, d1; oxaliplatin 100 mg/m^2^, iv over 2 h, d1; S-1 40 mg (BSA < 1.25 m^2^), 50 mg (BSA 1.25 ~ 1.5 m^2^) or 60 mg (BSA ≥ 1.5m^2^), po. bid, d1-14; every 3 weeks). Four cycles of DOS were administered from 6 to 8 weeks after surgery. The type of surgical procedure was determined by the location and extent of the primary tumor and was performed according to local standards. In terms of the metastatic lesions, surgeons evaluated the possibility of radical resection and whether the participants could tolerate the resection or not. The goal of surgery was a complete (R0 and at least D2) resection of the primary tumor, including standardized lymphadenectomy and, whenever possible, complete (R0) resection or complete macroscopic cytoreduction of the metastases.

Chemotherapy cohort: The participants were treated with eight cycles of DOS regimen followed by S-1 single-agent as maintenance therapy until PD (progressive disease). The DOS regimen was the same as that in the resection group.

The deescalated/modified DOS such as DO (Docetaxel and oxaliplatin) or SOX (Oxaliplatin and S-1) were allowed in this study if the investigator believed that it was in the best interest of the participant. In both cohorts, repeated imaging evaluations (CT/MRI of abdomen and pelvis, thoracic CT) were performed every 6 weeks during DOS chemotherapy and then every 2 months after that until PD, relapse, death, or the end of follow-up (November 2021). During the DOS treatment, the blood routine and biochemistry and physical examination were performed every 3 weeks before chemotherapy administration.

### Outcomes

The outcomes included OS, PFS and safety. OS was determined as the time interval from the beginning of DOS regimen to the date of death or last observation (censored). PFS was defined as the time interval from starting of DOS chemotherapy to (1) PD based on RECIST v1.1, (2) recurrence of the primary tumor or newly developed gastric adenocarcinoma, (3) distant metastasis, or (4) death. Adverse events (AEs) were classified by NCI-CTC AE (Version 4.0), and their relation to the treatment was judged by investigators. AEs were recorded until 28 days after the treatment ended.

### Statistical analysis

Continuous variables were presented as means ± standard deviation (SD) or median (interquartile range) and analyzed using Student’s t-test. Categorical data were presented as n (%) and compared by the Fisher’s exact test or the chi-square test. PFS and OS curves were generated using the Kaplan-Meier (K-M) method with Log-rank test. Effectiveness analysis was performed before and after propensity-score matching (PSM) based on age, gender, Lauren classification, location, metastasis, and HER-2 status. Cox proportional hazards model with 95% confidence interval (CI) was used to adjust for confounders (including gender, age, location, Lauren classification, metastasis, HER-2 status, First-line DOS cycles). *P* value < 0.05 is considered statistically significant.

## Results

### Baseline characteristics

**Fig. 1:**
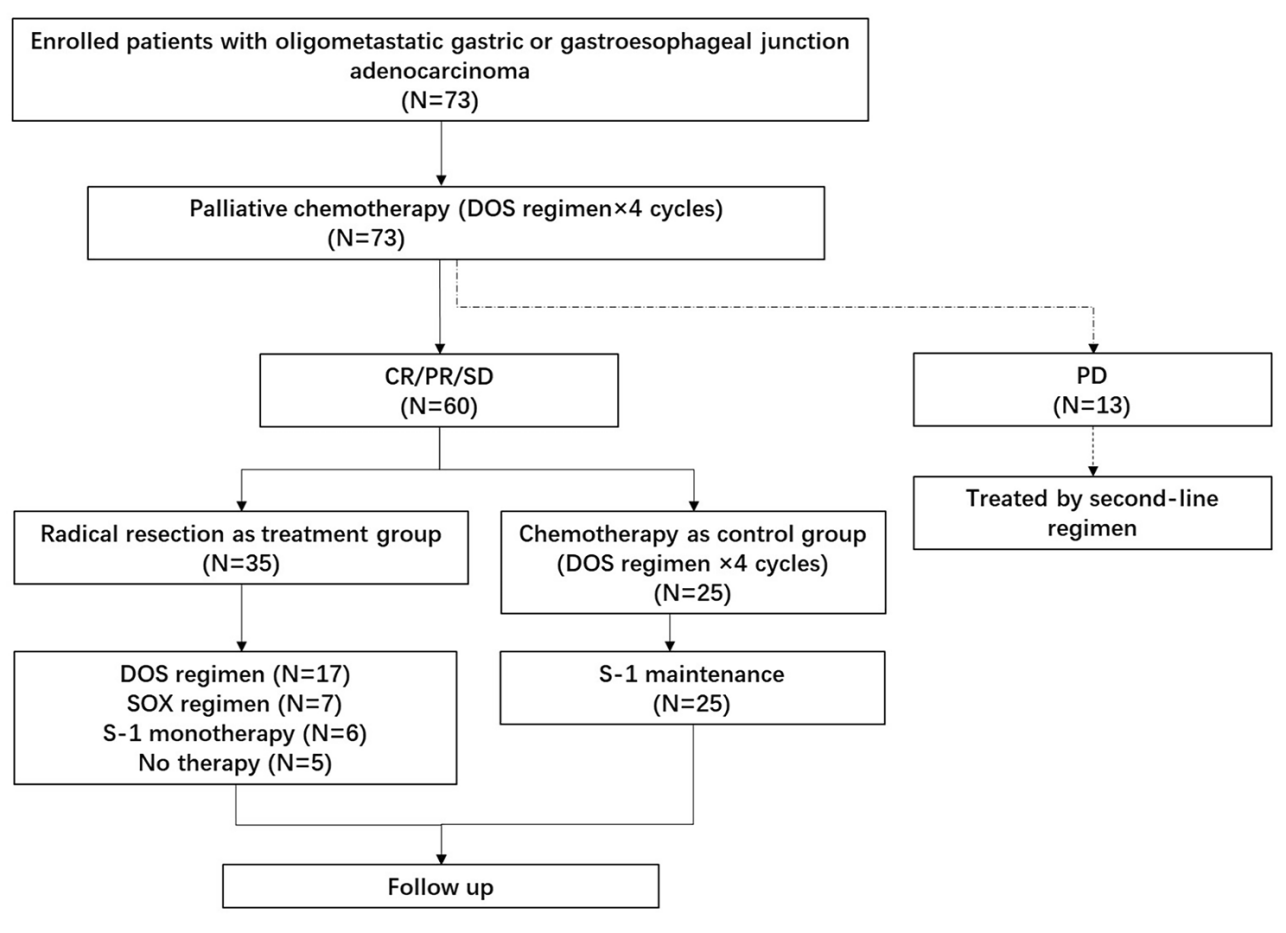
Flow chart of Neo-REGATTA study

Seventy-three gastric cancer patients with single non-curative factor were treated with DOS regimen. After four cycles, 13 patients got PD, and 60 patients were finally analyzed. Figure 1 presents the flowchart of this study. Among the 73 participants treated by DOS regimen as first-line chemotherapy, no complete remission (CR) was observed, 26 achieved partial remission (PR), 34 got stable disease (SD), and 13 patients got PD; therefore, the DCR (CR + PR + SD) was 82.2% (n = 60).

Before PSM, the median age of these patients with CR/PR/SD was 62.0 years. There were 47 male and 13 female patients. Thirty-five participants were in the resection group, and twenty-five participants were in the chemotherapy group. There were 10 patients with liver metastasis, 18 patients with lymph node metastasis, and 7 patients with peritoneal metastasis in the resection group. In the chemotherapy group, there were 6 patients with liver metastasis, 12 patients with lymph node metastasis, and 7 patient with peritoneal metastasis. Five participants (8.3%) had HER-2-positive disease among 60 patients, and they were treated with trastuzumab plus DOS regimen. After PSM, there were 19 participants in each group. There were no differences between the two groups regarding age, gender, location of the lesion, Lauren classification, metastatic site, HER-2, and DOS cycles before grouping (details shown in Table 1).

**Table 1:** Baseline characteristics of patients (before PSM and after PSM).

Characteristic	Before PSM			After PSM		
	Resection	Chemotherapy	P	Resection	Chemotherapy	P
n	35	25		19	19	
Gender, n (%)						
Female	11 (31.4)	2 (8.0)	0.064	2 (10.5)	2 (10.5)	1.000
Male	24 (68.6)	23 (92.0)		17 (89.5)	17 (89.5)	
Age, years, mean ± SD	59.0 ± 9.8	60.8 ± 9.8	0.475	61.2 ± 8.9	61.5 ± 8.6	0.927
Location, n (%)						
GEJ	9 (25.7)	7 (28.0)	> 0.999	7 (36.8)	8 (42.1)	> 0.999
non-GEJ	26 (74.3)	18 (72.0)		12 (63.2)	11 (57.9)	
Lauren classification, n (%)						
Intestinal	16 (45.7)	9 (36.0)	0.565	8 (42.1)	7 (36.8)	0.729
Diffuse	12 (34.3)	12 (48.0)		8 (42.1)	7 (36.8)	
Mixed	7 (20.0)	4 (16.0)		3 (15.8)	5 (26.3)	
Metastases, n (%)						
Liver metastasis	10 (28.6)	6 (24.0)	0.760	6 (31.6)	5 (26.3)	0.928
Lymph node metastasis	18 (51.4)	12 (48.0)		8 (42.1)	9 (47.4)	
Peritoneal metastasis	7 (20.0)	7 (28.0)		5 (26.3)	5 (26.3)	
HER-2, n (%)						
Positive	2 (5.7)	3 (12.0)	0.693	2 (10.5)	2 (10.5)	> 0.999
Negative	33 (94.3)	22 (88.0)		17 (89.5)	17 (89.5)	
DOS cycles before grouping, mean ± SD	3.71 ± 0.86	3.52 ± 0.96	0.415	3.58 ± 0.96	3.74 ± 1.10	0.640
Tumor response after 4 cycles of DOS						
CR	0	0	0.481	0	0	0.105
PR	17(48.6)	9(36.0)		12(63.2%)	7(36.8%)	
SD	18(51.4)	16(64.0)		7(36.8%)	12(63.2%)	

PSM: propensity score matching; SD: standard deviation; GEJ: gastroesophageal junction

### Treatment pattern

In the resection group, many patients (51.4%) could not endure intensive therapy after surgery. Seventeen participants (48.6%) still received the DOS regimen for a median of three cycles. Seven participants (20.0%) were treated with the SOX regimen for a median of two cycles. Six participants (17.1%) received S-1 monotherapy for half a year. Five participants (14.3%) could not endure postoperative therapy. There was no difference about PFS and OS if treated by individual regimen after radical resection. After surgery, there were 6 patients with T1, 11 patients with T2, 6 patients with T3, and 12 patients with T4a. About N stage, 20, 5, 3, 7 patients got N0, N1, N2, and N3, respectively. According to the tumor regression criterion of Japanese Gastric Cancer Association, 21 patients’ residual tumor was less than 1/3 of primary cancer, and the residual tumor accounted for 1/3 ~ 2/3 of primary cancer in 4 patients. 10 patients’ residual tumor was more than 2/3 of primary cancer. In the chemotherapy group, 25 patients were treated with the DOS regimen for a median of six cycles, then received the S-1 single-agent as maintenance until PD.

### Effectiveness before and after PSM

**Fig. 2:**
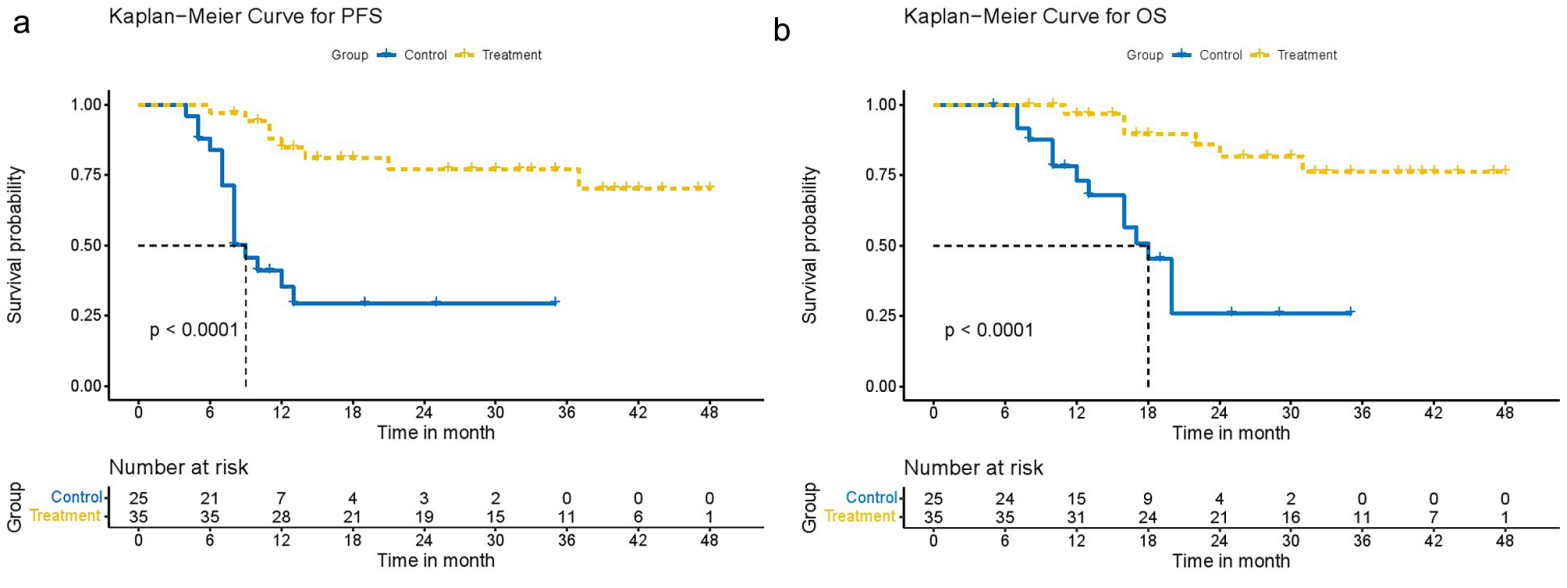
Kaplan-Meier curve for survival time in patients (Radical resection as treatment group; Chemotherapy only as control group). **(A)** PFS time for all patients. **(B)** OS time for all patients

After a median follow-up of 30.0 months (95% CI 23.5–36.5 months), the median PFS was 9.0 months (95% CI 7.0–11.0), and the median OS was 18.0 months (95% CI 15.1–20.9) for the chemotherapy group. The median PFS and OS were not reached in the resection group (Fig. 2). The rates of PFS at one-year and two-year were 35.3%, 29.1% in the chemotherapy group, and 84.7%, 77.1% in the resection group. The rates of OS at one-year and two-year were 73.1%, 25.8% in the chemotherapy group, and 96.9%, 81.7% in the resection group.

**Fig. 3:**
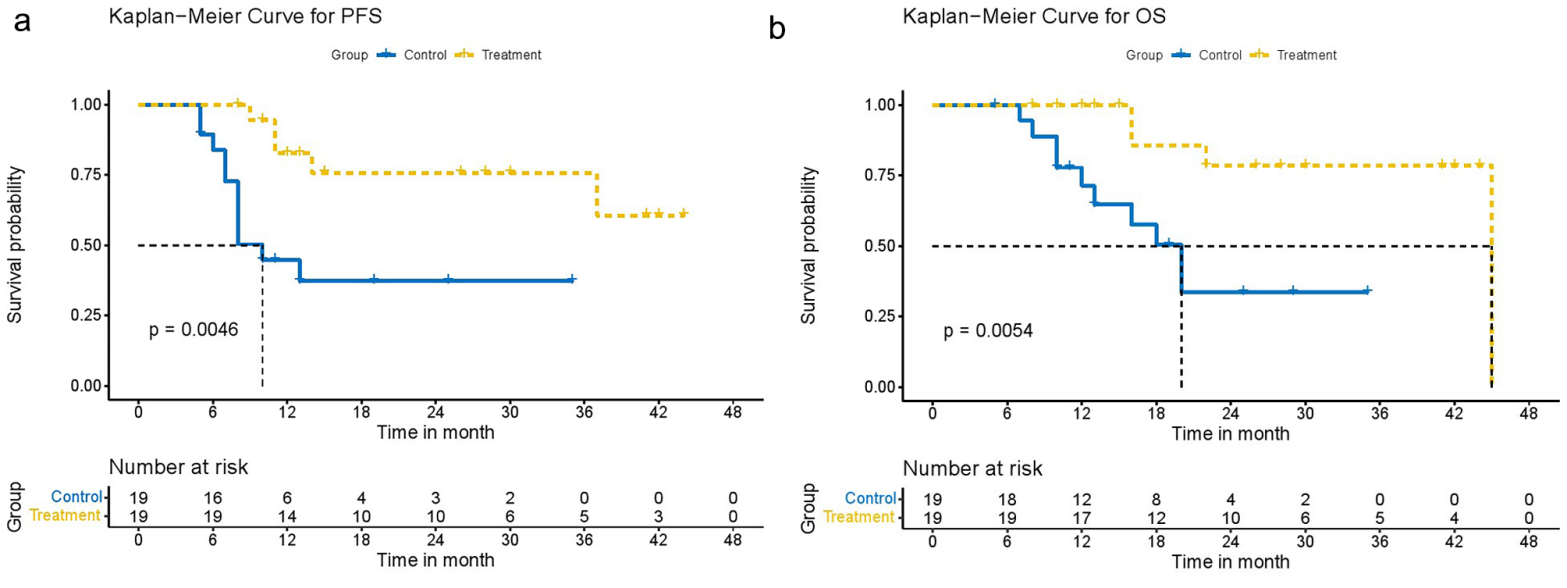
Kaplan-Meier curve for survival time in patients after PSM (Radical resection as treatment group; Chemotherapy only as control group). **(A)** PFS time after PSM. **(B)** OS time after PSM. PSM: propensity score matching

After PSM, the median OS was non-evaluable (NE) and 20.0 (95%CI 13.0-NE) months for the resection (n = 19) and chemotherapy (n = 19) groups, respectively (HR = 0.19, 95%CI: 0.05–0.70, P = 0.013). After PSM, the median PFS was non-evaluable (NE) and 10 (95%CI 8.0-NE) months for the resection group and the chemotherapy group (HR = 0.22, 95%CI 0.07–0.68, P = 0.009) (Fig. 3). The rates of PFS at one-year and two-year by propensity score analysis were 44.7%, 37.3% in the chemotherapy group, and 82.6%, 75.8% in the resection group.

### Adverse events

There were no severe surgical complications. In the resection group, the most common grade 1–2 AEs (> 10%) were neutropenia (25.7%), leukopenia (25.7%), and abnormal liver function (14.3%). The most common grade 3–4 AEs (> 5%) were neutropenia (5.7%) and leukopenia (5.7%). In the chemotherapy group, the most common grade 1–2 AEs (> 10%) were neutropenia (32.0%), leukopenia (32.0%), abnormal liver function (16.0%), and hand-foot syndrome (12.0%). The most common grade 3–4 AEs (> 5%) were neutropenia (8.0%) and leukopenia (8.0%) (Seen in Table 2).

**Table 2:** Treatment-related adverse events

	Resection (n = 35)		Chemotherapy (n = 25)	
AE, n (%)	Grade 1–2	Grade 3–4	Grade 1–2	Grade 3–4
Neutropenia	9 (25.7%)	2 (5.7%)	8(32.0%)	2 (8.0%)
Leucopenia	9 (25.7%)	2 (5.7%)	8(32.0%)	2 (8.0%)
Thrombocytopenia	3 (8.6%)	1 (2.8%)	2 (8.0%)	1 (4.0%)
Sensory neuropathy	2 (5.7%)	0	2 (7.1%)	0
Vomiting	1 (2.8%)	0	1 (4.0%)	0
Diarrhea	1 (2.8%)	0	1 (4.0%)	0
Fatigue	3 (8.6%)	1 (2.8%)	2 (8.0%)	1 (4.0%)
Abnormal liver function	5 (14.3%)	1 (2.8%)	4 (16.0%)	1 (4.0%)
Hand-foot syndrome	3 (8.6%)	1 (2.8%)	3 (12.0%)	1 (4.0%)
Stomatitis	2 (5.7%)	0	1 (4.0%)	0

AE: adverse events

## Discussion

Preoperative DOS regimen demonstrated promising anti-tumor effect and favorable safety profile in patients with advanced gastric cancer [26–28]. Whether radical resection after DOS treatment provide survival benefit compared with maintenance DOS in advanced gastric adenocarcinoma patients with a single non-curative factor remains unknown. The findings of this study showed that compared with continuous chemotherapy, radical resection might prolong the survival time in patients with single non-curable factor who had a disease control. In the resection group of this study, T stage, N stage, and the rate of residual tumor of primary gastric cancer were not associated with the survival time. We even found that the rate of pathological tumor regression was not consistent with the extent of tumor regression under CT scan.

Previous studies have shown that radical resection could benefit highly selected oligometastatic gastric cancer patients [29, 30]. Indeed, it has been reported that PFS of 25.5 months reached in 13 patients with metastatic disease (P1 or CY1) who achieved P0 or CY0 after chemotherapy and underwent R0 gastrectomy [31]. A SEER database study demonstrated radical resection of primary tumors and metastases is an independent factor associated with survival in gastric cancer cases [32]. Gastric cancer patients with limited metastasis in the AIO-FLOT3 trial (fluorouracil, leucovorin, oxaliplatin, and docetaxel regimen as preoperative treatment) highly benefited from neoadjuvant chemotherapy followed by surgery (median OS of 31.3 months) [33]. A retrospective analysis in gastric cancer patients with single non-resectable factor also indicated that, gastrectomy after chemotherapy could lead to survival benefit over palliative chemotherapy alone (after PSM, median OS: 15.9 vs. 10.0 months, P < 0.01) if the disease was controllable after chemotherapy [34]. However, the original REGATTA trial reported advanced gastric cancer patients with single non-curable factor administered D1 lymphadenectomy followed by palliative chemotherapy (S-1 + cisplatin) had no survival advantage compared to the continuous chemotherapy group, with median OS times of 14.3 months and 16.6 months, respectively [24]. In the surgery group of REGATTA trial, only primary gastric cancer was resected, but not the metastatic lesion. Therefore, we would investigate whether both primary and metastatic lesions received radical resection could improve the survival time and control the disease better. In this study, the median OS and PFS were significantly longer in radical resection group after DOS chemotherapy for 4 cycles than that in chemotherapy group, indicating that the survival time of patients with single non-curable factor might be prolonged by radical resection and DOS regimen.

As for safety, the incidence rates of grade 3 or 4 adverse effects in REGATTA trial, leucopenia (19% and 2%, respectively), anorexia (25% and 22%, respectively), nausea (15% and 5%, respectively), and hyponatremia (9% and 5%, respectively) were higher in patients received gastrectomy plus chemotherapy compared with chemotherapy alone [24]. In patients with advanced gastric cancer administered neoadjuvant DOS plus surgery or surgery and adjuvant S-1, the most common grade ≥ 3 AE was neutropenia (6.4% and 5.3%, respectively) [25]. In this study, the most common grade 3 or 4 adverse events (AEs) in the resection and chemotherapy groups were neutropenia (5.7% and 8.0%, respectively) and leukopenia (5.7% and 8.0%, respectively), which were similar between the two groups and the incidence rate was lower than that of REGATTA trial.

However, the performance status of some patients decreased after four-cycle DOS chemotherapy followed by radical resection of primary and metastatic lesions. They could not endure intensive chemotherapy after surgery, so only 17 patients (48.6%) still received DOS as postoperative regimen.

Therefore, future studies shall compare different perioperative chemotherapy regimens and include different types of patients to try to widen the indications of perioperative chemotherapy with radical resection of primary gastric cancer and oligometastasis. We also should explore the mechanism why some patients were sensitive, but others were resistant to chemotherapy, such as tumor microenvironment, immune escaping, or combining with target therapy, and so on. Also, we will design a complicated model for preoperative treatment to predict the efficacy and the prognosis of advanced gastric cancer.

## Conclusions

In conclusion, compared with continuous chemotherapy after four DOS cycles, radical resection (primary and oligometastasis) and adjuvant DOS led to better clinical outcomes in gastric adenocarcinoma patients with single non-curative factor without progression after the initial DOS treatment.

## Electronic supplementary material

Below is the link to the electronic supplementary material.

Supplementary Material 1

## Data Availability

The datasets used and/or analyzed during the current study are available from the corresponding author on reasonable request.

## Abbreviations

DOS: Docetaxel, oxaliplatin and S-1
DO: Docetaxel and oxaliplatin
SOX: Oxaliplatin and S-1
PSM: Propensity-score matching
