# Supplementary material for: Does resection after neoadjuvant chemotherapy of docetaxel, oxaliplatin, and S-1 (DOS regimen) benefit for gastric cancer patients with single non-curable factor? a multicenter, prospective cohort study (Neo-REGATTA)
Source: BMC Cancer. 2023 Apr 4;23:308. doi: 10.1186/s12885-023-10773-x (PMC10074668; doi:10.1186/s12885-023-10773-x)
Supplement: Supplementary file 1 — Supplementary Material 1 [file 12885_2023_10773_MOESM1_ESM.docx]

Title: Does resection after neoadjuvant chemotherapy of Docetaxel, oxaliplatin, and S-1 (DOS regimen) benefit for gastric cancer patients with single non-curable factor? A multicenter, prospective cohort study (Neo-REGATTA)

**The inclusion criteria were**: 1) 18-75 years of age, 2) Eastern Cooperative oncology Group (ECOG) performance status (PS) ≤1, 3) histologically confirmed gastric adenocarcinoma (including Lauren classification), 4) single oligometastasis revealed by thoracic CT, abdominal CT/magnetic resonance imaging (MRI) at first visit, including liver metastasis (H1), peritoneum metastasis (P1), para-aortic lymph node metastasis (No. 16), and ovarian metastasis, 5) measurable lesions according to RECIST v1.1 or peritoneum metastasis (P1) under laparoscopy, 6) performance status and organ function eligible for radical operation of primary and metastatic lesion, 7) baseline blood routine and biochemistry results in accord with following standards: hemoglobin ≥80 g/L, absolute neutrophil count (ANC) ≥1.5×10^9^/L, platelet ≥100×10^9^/L, alanine aminotransferase (ALT), aspartate aminotransferase (AST), alkaline phosphatase (ALP) ≤2.5 the upper limit of normal (ULN), total serum bilirubin (TSB) <1.5 ULN, serum creatinine (Scr) <ULN, and albumin (ALB) ≥30 g/L, 8) no severe concomitant disease leading to an OS of <5 years, and 9) ability to understand the study and/or comply with the protocol procedures.

**The exclusion criteria were:** 1) pregnant or lactating female, 2) positive hCG test in female of childbearing age, 3) patients with childbearing possibility who refused contraception, 4) previous treatment for gastric adenocarcinoma, including cytotoxic chemotherapy, radiotherapy, and immunotherapy, 5) other malignant disease in recent 5 years, excepting cured cutaneum carcinoma and cervical carcinoma in situ, 6) uncontrolled epilepsy, CNS disease or mental disorder that would probably affect the validation of informed consent and the obedience of taking medicine, 7) severe (active) heart disease, e.g., symptomatic cardiovascular disease (CVD), congestive heart failure (CHF) NYHA ≥1, arrhythmia in need of intervention, or myocardial infarction in the recent 12 months, 8) obstruction or dysfunction of upper GI tract or malabsorption syndrome which can affect the absorption of S-1, 9) preexisting peripheral neuropathy NCI CTC AE grade ≥1 (patients with only DTR will not be excluded), 10) organ transplant recipients who needed immunosuppression therapy, 11) severe uncontrolled recurrent infection, or other severe uncontrolled concomitant disease, 12) moderate to severe kidney injury (creatinine clearance rate (CCR) <50 ml/min, based on Cockroft and Gault formula) or Scr >ULN, 13) DPD deficiency patients, 14) allergic to platinum or any other drugs involved, or 15) having participated in other clinical trials in 4 weeks’ time before the enrollment.
